# Habitat–performance relationships of a large mammal on a predator‐free island dominated by humans

**DOI:** 10.1002/ece3.2594

**Published:** 2016-12-20

**Authors:** Andrew M. Allen, Augusta Dorey, Jonas Malmsten, Lars Edenius, Göran Ericsson, Navinder J. Singh

**Affiliations:** ^1^ Department of Wildlife, Fish and Environmental StudiesFaculty of Forest SciencesSwedish University of Agricultural SciencesUmeåSweden; ^2^ Division of BiologyImperial College LondonAscotBerkshireUK; ^3^ Department of Pathology and Wildlife DiseasesNational Veterinary Institute (SVA)UppsalaSweden

**Keywords:** fitness, habitat selection, Öland, parturition, performance, population dynamics, reproduction

## Abstract

The demographic consequences of changes in habitat use driven by human modification of landscape, and/or changes in climate, are important for any species. We investigated habitat–performance relationships in a declining island population of a large mammal, the moose (*Alces alces*), in an environment that is predator‐free but dominated by humans. We used a combination of demographic data, knowledge of habitat selection, and multiannual movement data of female moose (*n* = 17) to understand how space use patterns affect fecundity and calf survival. The calving rate was 0.64 and was similar to calving rates reported in other populations. Calf survival was 0.22 (annually) and 0.32 (postsummer), which are particularly low compared to other populations where postsummer survival is typically above 0.7. Home ranges were mainly composed of arable land (>40%), and selection for arable land was higher in winter than in summer, which contrasts with previous studies. Females that spent more time in broadleaf forest in the summer prior to the rut had higher fecundity rates, while more time spent in arable land resulted in lower fecundity rates. Females that spent more time in thicket/scrubland habitats during winter had lower calf survival, while females that had higher use of mixed forests tended to have higher calf survival. The dominance, and subsequent use, of suboptimal foraging habitats may lead to poor body condition of females at parturition, which may lower calf body weights and affect the mother's ability to lactate. In addition, our results indicated that the growing season has advanced significantly in recent decades, which may be causing a mismatch between parturition and optimal resource availability. These effects may exacerbate the female's ability to meet the energetic demands of lactation. Therefore, the observed low calf survival appears to be caused by a combination of factors related to current land use and may also be due to changing vegetation phenology. These results have important implications for the management of species in human‐dominated landscapes in the face of climate change, and for an increased understanding of how species may adapt to future land use and climate change.

## 1 Introduction

Studies of habitat–performance relationships (HPRs) aim to connect habitats with animal performance (Gaillard et al., 2010). Animal performance has been most commonly measured in terms of survival rates and reproductive success (Gaillard et al., 2010; McLoughlin et al., 2007; Van Moorter et al., 2009). Studies of HPRs have shown how animal performance may provide a better proxy of habitat quality than density, particularly for comparisons within populations (Gaillard et al., 2010; Mosser, Fryxell, Eberly, & Packer, 2009). For example, incorporating habitats in the home range that were important for forage and cover significantly affected the lifetime reproductive success of roe deer (*Capreolus capreolus*; McLoughlin et al., 2007). Studies of HPRs have also shown how ongoing changes in landscape structure and composition have impacts upon species and their population dynamics, with studies investigating how selected habitat influences survival and reproduction (Gaillard et al., 2010; Matthiopoulos, Fieberg, Aarts, Beyer, & Morales, 2015; McLoughlin et al., 2007; Pulliam & Danielson, 1991). For example, fragmentation of primary habitats may result in species utilizing suboptimal habitats in the matrix or restricting their movements, which may have negative impacts on reproduction or survival of young (Andren, 1994; Fischer & Lindenmayer, 2007; Fretwell & Lucas, 1969).

Investigating factors that influence survival of young may provide insights to how external factors influence recruitment and population growth rates (Aldridge & Boyce, 2008; Van Moorter et al., 2009). Large herbivores tend to display low fecundity and high adult survivorship (Gaillard, Festa‐Bianchet, Yoccoz, Loison, & Toïgo, 2000). Most large ungulates are capital breeders and use stored energy reserves to meet the demands of reproduction (Festa‐Bianchet, Gaillard, & Jorgenson, 1998; Jönsson, 1997; Stephens, Boyd, Mcnamara, & Houston, 2009). Calving rates, that is, the probability of a female having a calf, will be a function of the female's body condition at the time of ovulation (Milner, van Beest, Solberg, & Storaas, 2013; Sand, 1996) and the environmental conditions experienced during the gestation period (Gaillard, Festa‐Bianchet, Delorme, & Jorgenson, 2000; Milner et al., 2013). On the other hand, income breeders, such as roe deer, rely on energy acquired during the reproductive period (Andersen, Gaillard, Linnell, & Duncan, 2000; Jönsson, 1997). Due to the energetic costs of lactation, fewer differences are expected between capital and income breeders for postnatal care (Andersen et al., 2000). Neonate survival is highly dependent on climatic conditions, predation, population density, and the level of maternal care (Bårdsen et al., 2008; Gaillard, Festa‐Bianchet, & Yoccoz, 1998). The level of maternal care will depend upon the mother's experience and body condition (nutritional state), which may vary depending on environmental conditions and habitat quality (Parker, Barboza, & Gillingham, 2009; Sand, 1996; Senft et al., 1987). Poor nutritional state of females in late winter may have negative impacts on fetal development, resulting in lower calf body mass and potentially lower calf survival (Bowyer, Van Ballenberghe, & Kie, 1998; Keech et al., 2011; Sims, Elston, Larkahm, Nussy, & Albon, 2007). Following parturition, females in poor nutritional state may not meet the demands of lactation due to its high energetic costs (Cook et al., 2004; Milner et al., 2013; Parker et al., 2009). Conditions experienced during a calf's early development may have effects on future performance by influencing adult body size and future calf body size (Gaillard, Loison, Toïgo, Delorme, & Laere, 2003; Lindström, 1999).

Climatic factors may also have direct impacts on individual performance (Bårdsen et al., 2008). Warming temperatures have resulted in earlier plant growth in spring, resulting in a trophic mismatch between the timing of forage availability and parturition (Kerby & Post, 2013; Post, Pedersen, Wilmers, & Forchhammer, 2008). Increasing temperatures may induce heat stress in some species, which reduces the time available to feed and increases the energy required for body temperature regulation (Lenarz, Nelson, Schrage, & Edwards, 2009). Increasing winter temperatures may also be influencing fitness‐related traits, such as body weight (Forchhammer, Clutton‐Brock, Lindström, & Albon, 2001; Post, Stenseth, Langvatn, & Fromentin, 1997). Therefore, it is important to understand how human‐induced landscape change and the climate influence the performance of populations.

**Figure 1:**
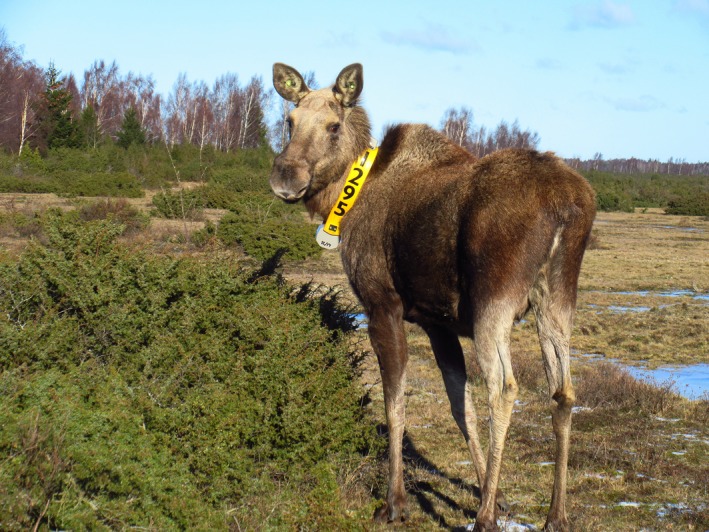
The study species, moose (*Alces alces*), wearing a GPS collar in a typical thicket‐type habitat on Öland. Photo: Fredrik Stenbacka

During recent years, moose (*Alces alces*; Figure 1) populations appear to be under environmental stress across their southern range (Lenarz et al., 2009; Monteith et al., 2015). A similar situation has been reported on the island of Öland in southern Sweden (Malmsten, 2014) with an average of 38 calves seen per 100 females (SAHWM, 2015). This is low when compared with nearby mainland Kalmar, with an average that rarely falls below 70 calves per 100 females (SAHWM, 2015). Postmortem examinations were previously conducted on a proportion of dead calves in Öland, which found the calves to be in subnormal body condition and starvation was the apparent cause of death (Malmsten, 2014). The postmortem came after a continued effort to restore the moose population, which has had limited success. The Öland moose population had previously been harvested at a constant rate during the 1990s, but in 2001 it became apparent that the population had fallen following mismanagement. A moratorium on hunting was introduced in an attempt to restore the population to previous levels. An aerial survey during 2005 indicated that the population was 150 with a density of 1.5 moose/10 km^2^ of suitable moose habitat (Jonsson 2007). Jonsson (2007) developed a population model that would allow a sustainable harvest and a continued population increase to 300 individuals. The model assumed a mean fecundity of 1.1 calves/female and natural calf mortality of 13%. However, it soon became apparent that recruitment was lower than expected, as evidenced by the low number of calves per female in the autumn, which may be due to either low fecundity, high calf mortality, or a combination of the two. The low productivity resulted in a steady decline in harvesting quotas, falling from a high of 41 harvested moose in the hunting season of 2009/10 to only six in 2014/15 (SAHWM, 2015). Understanding the causes for low recruitment would be imperative if a moose harvest were to continue on Öland. Furthermore, studying such populations can provide insights as to why populations are declining, particularly in the absence of hunting and predation.

Our study investigates habitat–performance relationships of moose on Öland. We relate the space use patterns within seasonal home ranges to performance, measured as fecundity and calf survival. We frame our analysis around two hypotheses about how habitat use may influence the performance of moose. Moose have been described as capital breeders, and thus rely on stored energy reserves for reproduction (Ericsson, Wallin, Ball, & Broberg, 2001; Milner et al., 2013). Therefore, calving rates are largely dependent on the female's body condition at the time of the rut. Furthermore, moose have higher flexibility in fecundity rates (number of calves per female) compared with many other ungulates. Moose can have a single calf or twins, and ovulation rate is related to body condition at the time of the rut (Franzmann & Schwartz, 1985; Keech et al., 2000). As the number of calves born to a female is dependent on the female's prewinter body condition (Testa & Adams, 1998), we would expect females that utilize preferential habitats such as broadleaf forest, clear‐felled areas, and young forests in the summer before the rut to have higher fecundity rates. The survival of calves however may be influenced by the female's space use patterns during both winter and summer. Adverse weather conditions or nonpreferential use of habitat such as agricultural areas and thicket‐type habitats during winter may lower the body condition of the female and reduce calf survival due to lower calf body weights, an inability to meet lactation demands, or the female prioritizing her own body reserves instead of lactation during the spring (Mathisen et al., 2010; Milner et al., 2013; Parker et al., 2009). Meanwhile, females that use preferential habitats after calving will better meet the demands of lactation and thus increase calf survival (Bowyer, Van Ballenberghe, Kie, & Maier, 1999).

To investigate how these factors may affect the performance of moose, our study aimed to answer the following questions:

1. How do females’ space use patterns during the summer before the rut influence fecundity rates?
2. How do females’ space use patterns during winter and summer affect calf survival?

We use a combination of movement, habitat use, and demographic datasets to explore these questions. We also explore climate trends over the last two decades. Understanding climatic effects on performance requires long‐term datasets of demography and climate, but unfortunately we have only recently begun measuring the demography of moose on Öland. Nonetheless, we describe climate trends on Öland and discuss how this may affect performance and recommend future avenues of research.

## 2 Materials and Methods

### 2.1 Study area

**Figure 2:**
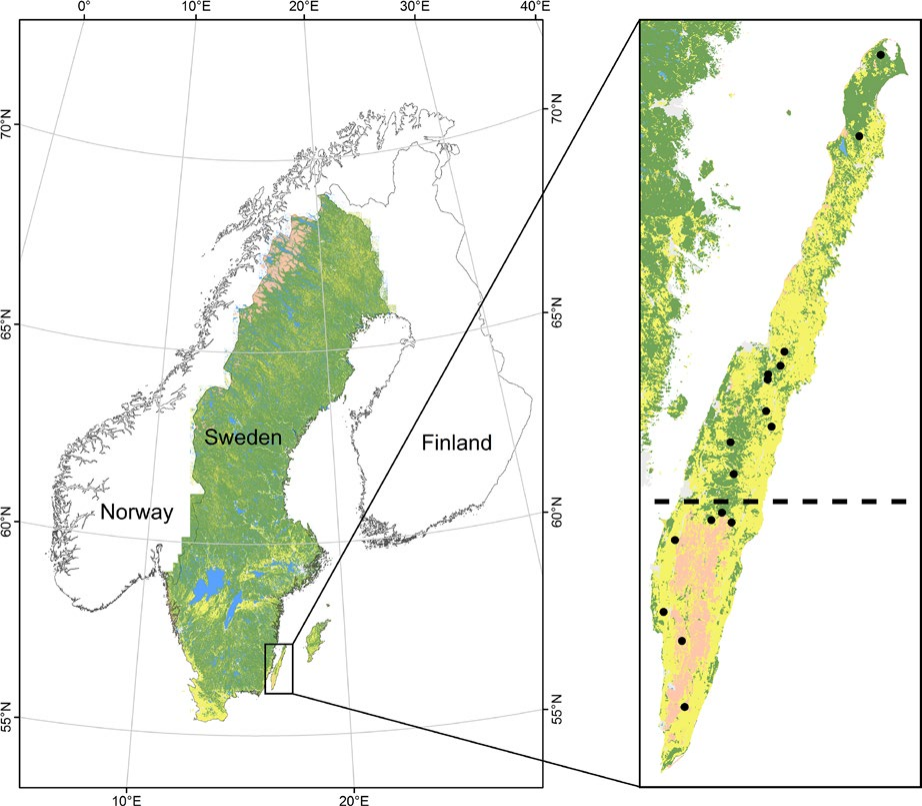
The location of the study area, Öland, including the coarse habitat structure of the island. In the right panel, yellow areas are arable land, green areas are forested, and the light pink area is the sparsely vegetated Stora Alvaret. The dashed line indicates the divide between the north and south, and the black dots are the average location for the females in the south (*n* = 7) and the north (*n* = 10)

Öland (56.748° N, 16.638° E), a Baltic island located in Kalmar county in southeast Sweden (Figure 2), is Sweden's second largest island (~1,342 km^2^ and 137 km in length). Öland has been intensively used for farming and livestock grazing (mostly cows and sheep) over the last centuries. Much of the wooded areas today originate from encroachment after cessation of grazing and abandonment of marginal farmland. The forests in the north have been heavily utilized by the timber industry for more than a century. The island is dominated by agricultural land (56%) followed by forest associated habitats (21%) and alvar grassland (19%). Alvars are found over dry, shallow, nutrient‐poor grazed soil on top of limestone bedrock, and a large alvar, the Stora Alvaret, is located in the southern part of Öland (~255 km^2^). The cessation of grazing and abandonment of farmland has resulted in the expansion of juniper (*Juniperus communis*) in the last century, especially in the last few decades (Bakker et al., 2012; Rosén, 2006). Although juniper is a native species, it can also be invasive and form thick impenetrable stands that outcompete other native vegetation, and for example, 55% of native plant species disappeared from juniper‐dominated plots (Bakker et al., 2012; Rosén & Bakker, 2005). The largest portions of forest habitats are in the northern (coniferous‐dominated) and central (deciduous‐dominated) parts of the island. Forest stands are dominated by birch (*Betula pubescens, B. pendula*) and Scots pine (*Pinus sylvestris*) with rowan (*Sorbus aucuparia*), aspen (*Populus tremula*), gray alder (*Alnus incana*), oak (*Qurecus rubra*), and willow sp. (*Salix* spp.) interspersed throughout. The field layer is primarily made up of bilberry (*Vaccinium myrtillus*), lowbush cranberry (*Vaccinium vitis‐id__a*), and heather (*Calluna vulgaris*). CORINE land cover data that document changes in land cover types were obtained for the period 2000–2006 and 2006–2012. During the 12‐year period, the largest changes were a net loss of 1.86 km^2^ of young forest, equating to 33% loss of the total coverage of young forest on the island. This corresponded with a net gain of 1.78 km^2^ of mature forest types.

Due to the large contrast in habitats between northern and southern Öland, we divided the study area into “north” and “south.” The extent of the Stora Alvaret was used to separate the north from the south. Both areas are dominated by agricultural land, but the Stora Alvaret forms the second dominant habitat in the south, whereas forest habitats are the second dominant habitat in the north (Figure 2). Roe deer also occur on the island, as do fallow deer (*Dama dama*), but the fallow deer are contained in an area at the southern tip of Öland. There are no red deer (*Cervus elaphus*). No accurate population figures are available for roe deer, but during the last 5 years the average number of harvested roe deer per 10 km^2^ was 22 on Öland compared with 7 on mainland Kalmar. There are no natural predators of moose on Öland. Moose hunting does occur, but in recent years this has been restricted to the southern part of the island, whereas roe deer are hunted on all of Öland. Moose on Öland are largely sedentary; however, a portion of tracked individuals have been found to migrate approximately 10 km between summer and winter home ranges (Allen et al., 2016).

### 2.2 Climate data

Temperature data were available from a weather station in central Öland (56°40′N, 16°27′E; SMHI 2015) and are summarized in Table 1. The study period was between 2012 and 2015, but climate data were obtained from the earliest date available, 1996, in order to detect climate trends during the previous 20 years. Precipitation data were available from another nearby weather station in central Öland (56°37′N, 16°40′E; SMHI, 2015) and are summarized in Table 1. The temperature data were used to estimate the start of the growing season, and mean temperatures were calculated for winter (December – March), spring (April/May, time of calving), summer (June–September), and autumn (October/November) from 1996 to 2015. The start of the growing season in Fennoscandia has been defined as the first day during which the mean daily temperature is above 7°C, and remains above 7°C for at least 2 weeks (Karlsen et al., 2007). A moving average of 7 days was applied to the data to remove brief peaks in temperature (Karlsen et al., 2007). Linear climate trends, for the start and end of the growing season and mean temperatures in winter, spring, summer, and autumn, were tested for significance using the Mann–Kendall trend test, which tests for trends in a time series based on the Kendall rank correlation (Mann, 1945).

**Table 1:** Summary of annual climate data for Öland during the 3 years of the study

Year	GS.St	GS.End	TempJu	TempJa	SnowD	Snow>5 cm
2012	28/04	26/10	17.1	0.94	11.9	76
2013	22/04	21/11	17.7	−0.92	6.4	6
2014	17/04	19/11	19.6	0.83	4.8	3

GS.St is the starting date of the growing season (dd/mm), GS.End is the end date of the growing season (dd/mm), TempJu is the mean daily temperature in the warmest month, July (°C), TempJa is the mean daily temperature in the coldest month, January (°C), SnowD is the mean snow depth (excluding days with no snow, measured in centimeters), and Snow>5 cm is the number of days that snow depth was >5 cm.

### 2.3 Movement data

Adult female moose (*n* = 20, $\overset{¯}{x}$ age = 7.5 years, range: 2–15 years) were immobilized (see Arnemo et al., 2006 for details) and equipped with global positioning system (GPS) neck collars (Vectronic Aerospace GmbH, Berlin, Germany) during the winter of 2011/12. The data were entered into the wireless remote animal monitoring (WRAM) database system for data validation and management (Dettki, Brode, Clegg, Giles, & Hallgren, 2014). The age of an individual was estimated by the tooth wear, a method commonly used for ungulates (Ericsson et al., 2001; Rolandsen et al. 2008). Females with <1 year of movement data were removed from the analysis, reducing the sample size to 17 individuals with ten in the north and seven in the south. The frequency of GPS positions ranged from every 30 min to 3 hr depending on season and sampling strategy. Therefore, all data were standardized to 3‐hr intervals (eight positions per day) using positions closest to midnight, 03:00, etc. The final dataset used for the estimation of home ranges and habitat selection consisted of 128,939 GPS positions. Data collection occurred between February 2012 and July 2015.

### 2.4 Home range/Utilization distribution

We identified seasonal home ranges, that is, summer and winter, in addition to annual home ranges. The start and end dates of the growing season (Table 1) were used to categorize GPS positions into either summer or winter home ranges, except for migratory moose (*n* = 2) where we used the dates that moose arrived at or left their seasonal home range. The dates of migration were determined using the net‐squared displacement approach (NSD; Börger & Fryxell, 2012) and fitted the models using the recommendations outlined in Singh, Allen, and Ericsson (2016). Home ranges were estimated using utilization distributions (UDs), more specifically the Biased Random Bridge (BRB) kernel approach (Benhamou, 2011) from the adehabitatHR package (Calenge, 2006). The diffusion coefficient was estimated from the data using the BRB.D function (Benhamou, 2011). The smoothing parameter (hmin) was estimated as the mean interlocation distance divided by 2 (Benhamou, 2011). The BRB approach estimates the UD over a grid, which can either vary in resolution among individuals or have a constant resolution if a grid is supplied. We created a grid with a 25 × 25 m resolution using the min/max XY coordinates of all individuals so that the resolution of the grid remained consistent between individuals and matched that of the habitat data (see Section 2.5 below). The 95% (UD95) isopleths were calculated from the UD, and each individual's UD was extracted for the habitat selection analyses. Variation in home range size was tested for significance using analysis of variance (ANOVA) between regions (north/south) and among years (2012–2015). Individuals with stable home ranges may have low interannual variability in calf survival if habitat is important. Therefore, we calculated the overlap of an individual's home range across seasons, that is, the overlap of winter home ranges and of summer homer ranges. We used the utilization distribution overlap index (UDOI) which has been shown to perform better when computing UD overlap (Fieberg & Kochanny, 2005).

### 2.5 Habitat selection

Land cover types were taken from the Swedish Land Cover Data map (from year 2003 at 25 m spatial resolution), and these were regarded as habitat types (Hagner, Nilsson, Reese, Egberth, & Olsson, 2005). The clear‐felled areas in the map were updated with latest data (2015) available from the Swedish Forestry Agency. Forests that had been clear‐felled were reclassified and aged where necessary into younger forest. The aging was performed according to forage availability for moose, where a clear‐fell was between 0 and 5 years and a young forest between 5 and 20 years (Månsson, 2007). The land cover map contained 60 classes, but these were condensed to 11 habitat type classes for further analysis. The habitats relevant for moose were reclassified by combining similar habitats; for example, the map contains six classes of coniferous forest which were combined into a single class for coniferous forest. The final habitat classifications included arable land, broadleaf forest, clear‐felled areas, coniferous forest, freshwater areas, thickets, mires, mixed forest, sparsely vegetated areas, urban areas, and young forest. Thickets are areas dominated by shrub species consisting of mostly juniper, *Rosa* spp., and *Crataegus* spp. The alvar grassland is primarily classified as thickets or sparsely vegetated areas. Young forests have no defined species proportions and typically have <70% of each species, whereas species proportions for a coniferous or broadleaf forest are >70% and a mixed forest range from 30% to 70% broadleaf forest (Hagner et al., 2005).

We analyzed habitat selection using a resource selection function (RSF; Manly, McDonald, Thomas, McDonald, & Erickson, 2007) with logistic regression that incorporated the mixed‐effect design of our study, that is, multiple observations per individual in the north and south. We investigated third‐order habitat selection, which compares habitats used to those available within the home range (Johnson, 1980; Thomas & Taylor, 1990). The RSF was estimated within a generalized linear mixed model (GLMM) using binomial logistic regression. We estimated the RSF for winter and summer separately and used the variable habitat type (with 11 habitat classes) as the fixed effect. Used habitats were those recorded at each GPS position. We generated five random positions for every GPS position to estimate available habitat. A circular buffer was created around every GPS position with a radius that captured 90% of distances travelled in a 3‐hr period (~415 m), and the random positions were drawn from this buffer (Boyce et al., 2003; Johnson, Parker, Heard, & Gillingham, 2002; Northrup, Hooten, Anderson, & Wittemyer, 2013). We excluded the final 10% when estimating the buffer in order to exclude exploratory and migratory movements. Logistic regression does not perform well when there are few cases of used or available habitats, for example, when habitats are used infrequently or not available (Gillingham & Parker, 2008). Therefore, for each individual, if either the used or available habitat was <5 points, we removed both the used and available points for that individual and habitat (Gillingham & Parker, 2008). After these changes, the freshwater habitat category was retained in the home range of only two individuals; therefore, we removed the category from the analysis. To capture variation in functional response (differential use of habitats), and variation between individuals, we included a random slope and a random intercept in the model (Gillies et al., 2006; Hebblewhite & Merrill, 2008). The random‐effects structure included individual nested within study region (north or south) to include variation among individuals and differences in habitat availability (Gillies et al., 2006).

### 2.6 Calf survival

The presence/absence of a calf, or twins, was recorded by tracking the cows at three separate occasions during each year following standard operational procedures (see Ericsson et al., 2001). The three occasions were (1) during the calving season (May and June), (2) before the annual moose hunt (late September), and (3) during late winter (February/March). Females were checked on several occasions during the calving season to accurately record the calving date in the field. In addition, GPS data were checked every 12 hr to determine whether a female's movement rate had declined and become confined to a limited area, which may indicate that a female is about to calve (Testa, Becker, & Lee, 2000a). The calf (or calves) was usually found within 3 days of birth. During the follow‐up checks, if the calf was not observed, additional checks were made to ensure we did not record a false mortality event. We estimated the calving rate, the proportion of females that have at least one calf, and fecundity, the number of calves born to a female. The survival trend was estimated using the Kaplan–Meier estimate (Kaplan & Meier, 1958). This method provides a survival function *S*(*t*), where *S* signifies the probability of an individual surviving until at least time t. A Fisher exact test was performed to determine whether females lactating in the spring experienced a reproductive cost the following autumn compared to females that did not lactate (Sand, 1998). Lactating females were those that still had a living calf after 4 weeks, whereas nonlactating females were those that either did not have a calf or lost their calf within the first 4 weeks.

To test our first question, we applied poisson regression to determine how the use of habitats during the summer prior to calving influenced the number of calves born the following spring, that is, fecundity. Therefore, we included habitats generally preferred by moose during summer. These habitats were broadleaf forest, clear‐felled areas, mires, young forest, and arable land (Bjørneraas et al., 2012; Månsson et al., 2015; Olsson, Cox, Larkin, Widén, & Olovsson, 2011). To test our second question, we applied binomial logistic regression to determine how a female's use of habitats influences calf survival. The response was females that had a calf that survived to the second check (i.e., autumn, *n* = 15) and those that lost their calf before the second check (*n* = 14). We considered three alternative hypotheses of how space use may affect calf survival. This included the use of nonpreferential habitats during winter (arable land, mires, sparsely vegetated areas, thickets, and urban areas), the use of preferential habitats during winter (broadleaf forest, coniferous forest, clear‐felled areas, mixed forest, and young forest), the use of preferential habitats during the summer after calving, and a combined model that incorporates all three alternative hypotheses. As some individuals had up to three observations, we considered a mixed‐effect model that included the ID of the individual as a random effect. The variance explained by the random effect was estimated to be zero given the data, suggesting that (1) the individual variance was very low, or (2) the data (i.e., low sample size) did not support estimating random variance among individuals, or a combination of both. Therefore, we used generalized linear models (GLMs) in the final model fitting process.

The explanatory variables were calculated by taking the proportion of GPS locations in a given habitat during winter or summer. Due to low sample size of females with calves (*n* = 29), we constrained the maximum number of variables to three to avoid overfitting the model. We display the top three models for each alternative hypothesis and also display the top three models that consider both habitat‐related and non‐habitat‐related factors that may influence calf survival by including the age of the female, twinning status (1 or 0), the average daily distance moved in winter, and the winter home range size. We also considered a separate model with year, and region (north or south), to investigate how these factors were related to calf survival. We used AICc (Burnham & Anderson, 1998) to determine the fixed‐effect structure of the model and variance inflation factors (VIFs) to check for colinearity and report the coefficient of determination, a type of pseudo‐*R* ^2^. As *R* ^2^ values are less than one for logistical regression, for clarity we report the modified *R* ^2^ which is adjusted to have a maximum of one (Nagelkerke, 1991). We used hierarchical partitioning (MacNally, 1996) to describe the importance of explanatory variables of the top three models in each alternative hypothesis in explaining variation in calf survival. Hierarchical partitioning has been described as the best way for identifying the most important variables from a set of already identified top models (Murray & Conner, 2009). All statistical analyses were performed in R (R Core Team 2013), the package lme4 (Bates, Maechler, Bolker, & Walker, 2014) was used to fit generalized linear models, and variable importance was measured with the hier.part function (Walsh & MacNally, 2013).

## 3 Results

### 3.1 Climate trends

The start of the growing season advanced significantly between 1996 and 2015 (Mann–Kendall: *p* = .005; Appendix S1). The mean start date of the growing season had advanced by more than 2 weeks when comparing the 5‐year mean between 1996 and 2000 ($\overset{¯}{x}$ = 09 May, SD = 17.3 days) and 2011 and 2015 ($\overset{¯}{x}$ = 19 April, SD = 6 days). Average monthly temperatures in winter were quite variable across years (January: $\overset{¯}{x}$ = 0.25, SD = 1.94; May: $\overset{¯}{x}$ = 10.36, SD = 1.41; July: $\overset{¯}{x}$ = 17.64, SD = 1.57; Appendix S1). There was no significant trend in temperatures in winter (*p* = .627, Mann–Kendall test), summer (*p* = .922), or autumn (*p* = .230), but there was a trend of increasing spring temperatures (*p* = .010; Appendix S1).

### 3.2 Home range/Utilization distribution

A total of 135 home ranges were estimated, consisting of 45 annual, summer, and winter home ranges (north = 28, south = 17). The mean annual HR for moose on Öland was 12.47 km^2^ (SD = 3.80 km^2^) with a mean summer HR of 9.41 km^2^ (SD = 3.23 km^2^) and a mean winter HR of 9.23 km^2^ (SD = 3.63 km^2^). Annual (north = 12.73 km^2^, south = 12.04 km^2^, *F*_[1,43]_ = 0.34, *p* = .561) and summer home ranges (north = 9.47 km^2^, south = 9.31 km^2^, *F*_[1,43]_ = 0.11, *p* = .738) were similar in size across areas, but winter HRs were larger in the north (north = 10.49 km^2^, south = 7.14 km^2^, *F*_[1,43]_ = 11.09, *p* = .002). Among years, the winter HR was larger than the summer HR in the north (*p* = .048) during 2012/13. Home ranges showed relatively high overlap during summer ($\overset{¯}{x}$ = 0.82, SD = 0.32) but more so in the north ($\overset{¯}{x}$ = 0.96, SD = 0.18) than in the south ($\overset{¯}{x}$ = 0.61, SD = 0.38). Home ranges showed less overlap during winter though ($\overset{¯}{x}$ = 0.50, SD = 0.12) with similar levels of overlap in the north ($\overset{¯}{x}$ = 0.49, SD = 0.12) and the south ($\overset{¯}{x}$ = 0.52, SD = 0.25).

### 3.3 Habitat selection

Home ranges of individuals in both regions were dominated by arable land all year round (Appendix S2). The proportion of arable land in the home range did not vary between females with or without calves in neither the north (*p* = .951) nor south (*p* = .463). After arable land, the dominant habitats in home ranges in the north were broadleaf forests and young forests. After arable land, the dominant habitat in home ranges of individuals in the south was sparsely vegetated areas (Appendix S2).

Habitat selection within home ranges was variable across seasons and years (Table 2). Selection for arable land increased during winter, and only broadleaf forest, thickets, and young forest were significantly selected over arable land. Clear‐felled areas ranked highest in summer followed by broadleaf forest (Table 2). Selection for thickets changed substantially between summer (ranked 5th) and winter (ranked 1st). Urban areas, sparsely vegetated areas, and mires were ranked lowest (Table 2).

**Table 2:** Odds ratio results and habitat rankings for the resource selection function for summer (top) and winter (bottom). Model results were obtained by manually setting a different habitat category as the intercept. Each column represents the habitat type that was set as the intercept. The values listed within each column are the odds ratios of the remaining habitats in comparison with the intercept (which are highlighted in bold)

Habitat	Rank	AL	BF	CL	CF	MI	MF	SV	TH	UR	YF
Summer											
AL	8	**0.139** **	0.456**	0.441*	0.570**	0.619*	0.509**	1.638	0.566**	2.576**	0.508**
BF	2	2.212**	**0.305** **	0.967	1.250*	1.353	1.116	3.626*	1.244	5.632**	1.113**
CL	1	3.000*	1.348	**0.316** **	1.651	1.853	1.461	4.910*	1.689	7.521**	1.517
CF	6	1.788**	0.803*	0.777	**0.244** **	1.086	0.893	2.975*	1.000	4.526**	0.893
MI	7	1.612*	0.732	0.708	0.913	**0.225** **	0.812	2.546	0.908	4.176**	0.808
MF	3	2.010**	0.901	0.872	1.128	1.219	**0.273** **	3.347*	1.123	5.063**	1.005
SV	9	0.820	0.365*	0.367	0.466*	0.489	0.410	**0.085** **	0.452*	2.131	0.401*
TH	5	1.796**	0.806	0.788	1.020	1.087	0.908	2.839	**0.246** **	4.582**	0.900
UR	10	0.393**	0.176**	0.166**	0.223**	0.237**	0.196**	0.634	0.218**	**0.054** **	0.195
YF	4	1.979**	0.899**	0.869	1.123	1.215	1.003	3.257*	1.117	5.058**	**0.274** **
Winter											
AL	6	**0.193** **	0.767*	0.985	0.939	2.297**	0.774	2.38**	0.701**	2.895**	0.771*
BF	2	1.303*	**0.251** **	1.277	1.224*	2.992**	1.008	3.100**	0.913	3.769**	1.005
CL	7	0.911	0.698	**0.195** **	0.854	2.080	0.703	2.167*	0.638	2.647**	0.701
CF	5	1.064	0.816*	1.045	**0.205** **	2.442**	0.823*	2.53**	0.746	3.083**	0.820*
MI	8	0.433**	0.332**	0.408**	0.407**	**0.084** **	0.335**	1.030	0.303**	1.249	0.334**
MF	4	1.292	0.992	1.283	1.215*	2.967**	**0.249** **	3.074**	0.906	3.747**	0.997
SV	9	0.421**	0.323**	0.422**	0.395**	0.962	0.325**	**0.081** **	0.295**	1.223	0.324**
TH	1	1.427**	1.095	1.409	1.339	3.274**	1.103	3.395**	**0.275** **	4.126**	1.100
UR	10	0.347**	0.266**	0.344**	0.325**	0.795	0.268**	0.825	0.243**	**0.067** **	0.267**
YF	3	1.297*	0.995	1.268	1.219*	2.979**	1.004	3.086**	0.909	3.752**	**0.250** **

AL, arable land; BF, broadleaf forest; CL, clear‐felled area; CF, coniferous forest; MI, mires; MF, mixed forest; SV, sparsely vegetated area; TH, thickets; UR, urban area; YF, young forest.

The odds ratios indicate preference (>1) and avoidance (<1) compared to the intercept habitat.

Significance is shown as *(*p* < .05) and **(*p* < .01).

### 3.4 Calf survival

During the 3 years of the study, there were 16 occasions that a female did not reproduce, while 29 calving events were recorded (Appendix S3: Figure S1). The calving rate (the probability of a female having a calf) was 0.64 (north = 0.67, south = 0.58). The 29 calving events consisted of 14 singletons and 15 twins; thus, the total number of calves born was 44. The mean calving date was the 15th of May for both the north (SE = 1.81) and the south (SE = 2.41). The rate of twinning was higher in the north (63%) than in the south (30%); however, the twinning rates varied in the north with higher rates in 2012 and 2013 and relatively low in 2014 (Appendix S3: Figure S1). The mean age of females that did not reproduce was 7.3 (SD = 2.5, range = 2–15), similar to the mean age of all females ($\overset{¯}{x}$ = 7.8, SD = 4.1). Four females did not reproduce during the entire study, two from the north that were aged 5 and 9 at the start of the study and two from the south that were aged 2 and 15 at the start of the study (Appendix S3: Figure S1), although one female was only tracked for 1 year compared to the others that were tracked for 3 years. Fecundity was variable across years for females that did reproduce, with only five females producing at least one calf every year (north = 4, south = 1; Appendix S3: Figure S1). The top model explaining fecundity rates showed a positive relationship between time spent in broadleaf forest and the number of calves born the following spring (*p* = .040; Appendix S3: Figure S2; Appendix S3: Table S1). The next best model found a negative relationship between time spent in arable land and fecundity (*p* = .051, ΔAIC = 0.28; Appendix S3: Figure S2; Appendix S3: Table S1). A Fisher's exact test showed that calving rates of females did not significantly differ between females that had lactated (*n* = 13) or not (*n* = 9), the previous spring (lactating = 0.769, nonlactating = 0.777, *p* = 1.000).

**Figure 3:**
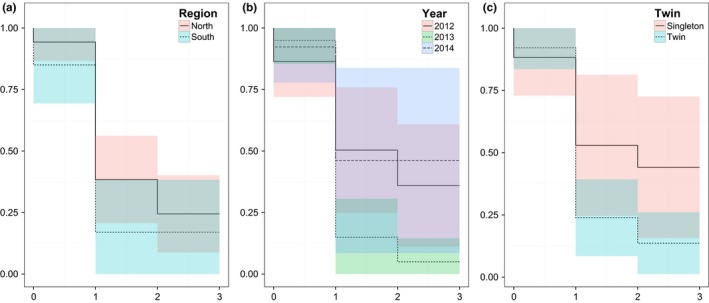
Kaplan–Meier estimates of moose calf survival: (a) survival estimates for the north and the south of Öland; (b) survival estimates during the 3 years of the study, 2012, 2013, and 2014; and (c) survival estimates of calves that are either singletons or twins. The lines indicate the Kaplan–Meier estimate at each time step, and the shaded areas are the 95% confidence intervals. Time step 0 is the birth of the calf, 1 is the start of summer, 2 is after summer but before the hunt, and 3 is late winter

The Kaplan–Meier estimate for the study period indicated that mean calf survival was 0.91 (SE = 0.04) at the start of summer, 0.32 (SE = 0.07) at the end of summer but before the hunt, and 0.22 (SE = 0.06) in late winter. The majority of mortality events occurred during the summer, that is, between the first check performed at the start of summer and the second check performed before the hunt in early autumn (Figure 3). The low survival meant that recruitment (calves per cow in the autumn before the hunt) was 0.20 (north = 0.27, south = 0.06). Calf survival was low in both the north ($\overset{¯}{x}$ = 0.24, SE = 0.08) and the south ($\overset{¯}{x}$ = 0.17, SE = 0.11; Figure 3a). There was variation in calf survival among years with a low of 0.05 (SE = 0.05) in 2013 compared to 0.36 (SE = 0.13) in 2012 and 0.46 (SE = 0.19) in 2014 (Figure 3b). The majority of calves were twins (*n* = 30 of a total 44 calves), but survival of twins was lower, 0.14 (SE = 0.06) compared to singleton calves with survival of 0.44 (SE = 0.15; Figure 3c). A Fisher's exact test showed that survival of calves did not significantly differ depending on whether females had lactated (*n* = 10) or not (*n* = 7), the previous spring (lactating = 0.300, nonlactating = 0.571, *p* = .350).

**Figure 4:**
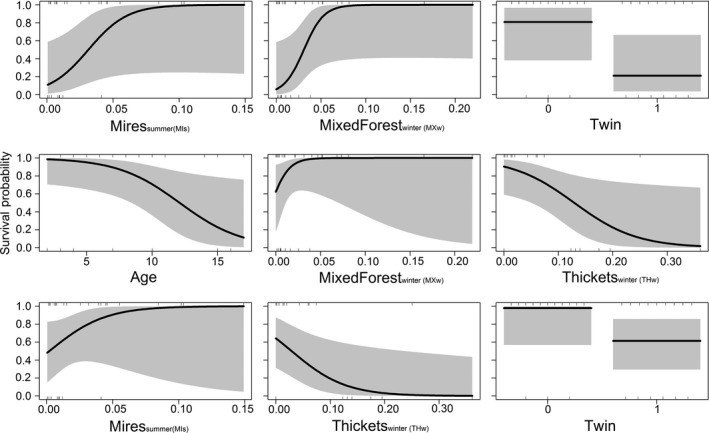
Modeled relationships and uncertainty of the top three models containing the response (calf survival) and explanatory variables of (a) Mires (MIs) + Mixed Forest (MXw) and Twin; (b) Age + MXw + Thickets (THw); and (c) MIs + THw + Twin. “w” and “s” indicate the proportion of time spent in a habitat during winter (w) or summer (s)

**Figure 5:**
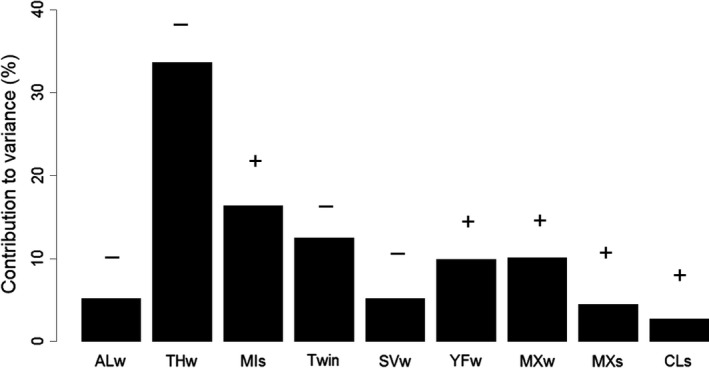
Results of hierarchical partitioning to measure the importance of explanatory variables included in Table 3. The percentage indicates the total contribution of a variable toward explained variation. AL, arable land; CL, clear‐felled area; MI, mire; MX, mixed forest; SV, sparsely vegetated area; THw, thickets; YF, young forest; w, winter; and s, summer. + and − indicate the direction of the relationship

Only two females had a calf alive in autumn every year, while no female had 100% mortality of calves during summer. The large within‐individual variation in calf survival across years may explain why including ID as a random effect did not improve model fit. Considering the three individual alternative hypotheses, the use of nonpreferential habitats provided the best model fit and explained most variation in calf survival (Table 3). In particular, the use of thickets during winter had a negative effect on calf survival. The combined models provided significantly better fit (i.e., ΔAIC < 4) than the alternative hypotheses. The top model explained 62% of variation in calf survival where the use of mixed forest during winter (MXw) and mires during summer (MIs) had a positive effect on survival (Table 3; Figure 4). The twinning status was included in two of the combined models with twins having lower survival, and similarly, survival decreased as individuals aged (Table 3; Figure 4). The use of thickets during winter (THw) was again important and included in two of the top three models with survival decreasing as the use of thicket habitats increased (Table 3; Figure 4). In addition, hierarchical partitioning indicated that THw explained most variation when considering all variables listed in Table 3 (Figure 5). Region was not a significant factor in explaining calf survival, whereas there was variation in survival across years, with 2013 having significantly lower survival than 2012 and 2014 (Table 3; Appendix S4). Model‐averaged coefficients and the relative variable importance for all variables listed in Table 3 are shown in Appendix S4.

**Table 3:** Results for the top logistical regression models explaining variation in calf survival on Öland

Model	*df*	Residual	AICc	ΔAICc	*W*_i_	*r* ^2^
Nonpreferred Winter Habitat						
THw	27	28.51	32.97	4.19	0.03	.32
ALw + THw	26	26.26	33.23	4.45	0.03	.40
ALw + SVw + THw	25	23.92	33.59	4.81	0.02	.48
Preferred Winter Habitat						
MXw	27	30.90	35.36	6.58	0.01	.22
YFw	27	31.09	35.55	6.77	0.01	.22
MXw + YFw	27	28.90	35.8.6	7.08	0.01	.30
Preferred Summer Habitat						
MIs	27	29.53	33.99	5.21	0.02	.28
MIs + MXs	26	27.38	34.33	5.55	0.02	.36
CLs + MIs + MXs	25	25.30	34.97	6.19	0.01	.43
Combined Models						
MIs + MXw + Twin	25	19.12	28.78	0.00	0.27	.62
Age + MXw + THw	25	19.66	29.33	0.55	0.21	.60
MIs + THw + Twin	25	19.72	29.38	0.60	0.20	.60
Alternative Models						
Region	27	33.42	37.88	9.10	0.00	.12
Year	27	23.03	29.99	1.21	0.15	.51
NULL	28	35.92	38.07	9.29	0.00	.00

W, winter; s, summer. Age, age of the individual; AL, arable land; CL, clear‐felled area; MI, mire; MX, mixed forest; SV, sparsely vegetated area; TH, thickets; Twin, twinning status (0,1); Region, north or south; and Year, year of data collection (2012, 2013, 2014).

*W*_i_ is the Akaike weight of all models listed in the table, and *r* ^2^ is the modified coefficient of determination.

## 4 Discussion

Summer calf survival on Öland during the study period was 0.32, and annual survival was 0.22. This is particularly low when compared to other Fennoscandian populations where summer survival rates have been reported to be in excess of 0.80 in northern Sweden (Ericsson et al., 2001), 0.64 in a population exposed to brown bear (*Ursus arctos*) predation in central Sweden (Swenson et al., 2007), and between 0.68 and 0.93 in Norwegian populations (Milner et al., 2013; Stubsjøen, Sæther, Solberg, Heim, & Rolandsen, 2000). Calf survival on Öland more closely resembles survival rates in Alaska where the majority of mortality events were due to predation (Ballard, Whitman, & Reed, 1991; Testa, Becker, & Lee, 2000b). A number of factors other than predation have been shown to affect calves’ survival during summer, such as climatic variation, heat stress, malnutrition, abandonment, disease, and poor maternal investment (Gaillard, Festa‐Bianchet, Delorme et al., 2000; Lenarz, Fieberg, Schrage, & Edwards, 2010; Monteith et al., 2015; Post et al., 2008). Furthermore, low survival in a single year has been attributed to adverse weather conditions, high population density, and low food quality (Peterson, 1999; Stubsjøen et al., 2000). Given that our results indicate that calf survival was consistently low, as opposed to a single year event, and together with the results of the postmortem described in the introduction, the performance of moose on Öland appears to be influenced by food‐related environmental and/or climatic factors.

Large‐bodied herbivores such as moose are expected to invest in reproduction in accordance with their nutritional state (Monteith et al., 2013, 2015; Parker et al., 2009). The calving and twinning rates on Öland were similar to other populations (Gingras, Couturier, Côté, & Tremblay, 2014; Milner et al., 2013; Testa, 2004; Testa & Adams, 1998), which indicates that females did not have poor prewinter body condition and thus did not encounter a trade‐off between reproduction and accumulating fat reserves (Monteith et al., 2013, 2015). Our results provide additional support for how a female's space use patterns during summer influence fecundity, with females spending more time in preferential habitats such as broadleaf forest having higher fecundity rates. Females’ space use was consistent across summers indicating that resources were stable across years. In addition, females that had lactated in the spring were able to attain a body condition that allowed them to reproduce again the following spring, indicating there was little carryover effect of lactation (Sand, 1998).

Our results provide evidence for a habitat–performance relationship and that the environmental conditions during the winter before calving had a strong influence on calf survival. Increasing use of thickets during winter appeared to negatively affect calf survival. Habitat selection for thickets was ranked highest during winter (Table 2), but the use of thickets explained the most variation in calf survival (Figure 4). Thickets, consisting of scrubland and woody vegetation, would not be considered an optimal foraging habitat for moose. Moose are normally associated with forest habitats (Bergström & Hjeljord, 1987; Bjørneraas et al., 2011), but the availability of suitable foraging habitats may be limited, as most home ranges were dominated by arable land. Arable land may provide good foraging opportunities during summer and autumn, such as oat crops or by creating edge habitats such as field margins with forest (Bjørneraas et al., 2011; Månsson et al., 2015). However, in Norway, they have been described as poor foraging habitats during winter and only fair during spring (Bjørneraas et al., 2011). The coverage of thickets, especially juniper, has also expanded dramatically in recent decades due to agricultural abandonment and cessation of grazing, and restoration efforts are being made to control the spread (Bakker et al., 2012; Rosén & Bakker, 2005). Although juniper has previously been described as a preferred forage item for moose (Månsson, Kalén, Kjellander, Andrén, & Smith, 2007; Shipley, Blomquist, & Danell, 1998), the abundance of juniper in these areas was relatively low. It is uncertain whether a diet dominated by juniper may meet the nutritional demands of moose (Felton et al., 2016; Ohlson, Staaland, Ohison, & Staaland, 2001), and further research would be needed. The spread of juniper results in the loss of native vegetation (Rosén, 2006), which may also restrict foraging opportunities for moose. Future research that compares space use patterns between restored areas where juniper has been removed and juniper‐dominated areas would clarify whether the spread of juniper is affecting forage availability. Our results indicate that the lack of suitable foraging habitats associated with forest during winter may push females into suboptimal habitats such as thickets and arable land, meaning that females have poor body conditions at the end of winter, which has subsequent negative impacts on fetal development and the level of maternal care provided after birth (Parker et al., 2009; Sims et al., 2007).

Calf survival was higher for females that had higher selection for mires and clear‐cut areas during summer. Lactation after birth has high energetic demands (Bowyer et al., 1998; Sand, 1998), and to meet the demands of lactation, parturition is closely linked to the start of the growing season when forage quality is highest (Hebblewhite, Merrill, & McDermid, 2008; Monteith et al., 2015). New vegetation growth in mixed forest and clear‐cut areas has high nutritional value (Wam, Histøl, Nybakken, Solberg, & Hjeljord, 2016), and thus, it is perhaps not surprising that increased use of these habitats resulted in better performance. It is interesting to note however that increasing use of mire habitats resulted in higher calf survival. Mires are not traditionally considered as favorable habitats for moose (Bergström & Hjeljord, 1987; Olsson et al., 2011); however, Bjørneraas et al. (2012) found that females with calves spent more time in mire habitat than expected by chance (and compared to males and females without calves), indicating that mire habitats may hold some nutritive value for females with calves.

Other factors that were not measured during this study may affect the performance of moose on Öland and require further investigation. We lacked information on population density and thus could not study how density dependence may influence habitat–performance relationships. Studies have shown that recruitment rates may fall as densities become very high (Fryxell, Mercer, & Gellately, 1988; Gaillard et al., 1998), although moose may be more resilient to density effects by adjusting their reproductive strategy (Gingras et al., 2014; Grøtan, Sæther, Lillegård, Solberg, & Engen, 2009). The population size of moose on Öland has been described as low compared to previous levels, but the density may be high considering the levels of suitable habitat. This may explain why moose utilize suboptimal habitats such as thickets during winter, and the subsequent competition for resources, particularly if the suitability of these habitats has degraded following the expansion of juniper (Rosén & Bakker, 2005). It is also unclear whether the high density of roe deer may exacerbate inter‐ and intraspecific competition for resources, especially considering the relatively large overlap in diet (>50% in main plant groups; Mysterud, 2000). The low population size, isolation from the mainland, and former harvesting protocols such as age‐ and sex‐biased hunting may also raise concerns about inbreeding depression (Hedrick & Kalinowski, 2000; Herfindal et al., 2014). Inbreeding depression may influence calf body mass and twinning rates (Haanes et al., 2013), and understanding these effects would require further research. Fortunately, understanding genetic diversity is becoming increasingly feasible as technologies become cheaper due to advances such as next‐generation sequencing (McCormack, Hird, Zellmer, Carstens, & Brumfield, 2013) and samples more readily available through techniques such as noninvasive sampling (Norman & Spong, 2015).

In addition to the effects of habitat quality, it is important to consider how climate may influence the performance of females, such as variability in the timing and the rate of green‐up (Pettorelli, Pelletier, von Hardenberg, Festa‐Bianchet, & Côté, 2007). We observed a significant advance in the start of the growing season, which is supported by a significant increase in spring temperatures (Appendix S1). In addition, we found that the date of parturition was on average 3 weeks later than the start of the growing season during this study. Unfortunately, we do not have longitudinal data on parturition rates; however, Bowyer et al. (1998) found that the timing of parturition was not directly related to conditions during winter or spring, but instead that the timing of parturition was adapted to long‐term patterns of climate. The earlier onset of the growing season means that the period of highest forage quality may have already passed when the energetic demands of lactation are greatest. Further research, including longitudinal studies, would be needed to investigate how the rate of green‐up on Öland may influence the performance of individuals (Monteith et al., 2015; Pettorelli et al., 2007).

Other climatic effects that need consideration are temperature. Warmer temperatures increase the persistence of disease and parasites in the environment. An increasing number of disease‐related issues have been reported in North America (Murray et al., 2006), and traces of the tick‐borne *Anaplasma phagocytophilum* have been found on moose in Öland (Malmsten et al., 2014). Other temperature effects include moose becoming heat‐stressed at −5°C during winter and 14°C during summer, although habitat use patterns may only change at thresholds above 20°C (van Beest, Van Moorter, & Milner, 2012; Melin et al., 2014). Summer temperatures were below the 20°C threshold, but the average daily temperature during the coldest month, January, was 0.7°C, which is nearly 6°C warmer than the winter threshold. Questions have been raised about the threshold levels (van Beest et al., 2012; Lowe, Patterson, & Schaefer, 2010; Melin et al., 2014), and further research is needed to understand these effects. However, warm winter temperatures may induce heat stress and increase the energy required for thermoregulation (Lenarz et al., 2009). The combined effects of heat stress and poor foraging opportunities may have cumulative negative impacts on the postwinter body condition of females.

In conclusion, our results provide strong evidence for habitat–performance relationships of moose on Öland. The dominance of arable land, and subsequent lack of foraging opportunities in optimal or high quality habitats, may force females to use suboptimal habitats, especially during winter. For example, thickets were the preferred habitat during winter even though increasing use of thickets was associated with lower calf survival. This may lead to poor postwinter body condition which influences the body mass of calves at birth and maternal care provided after birth. As Öland is a predator‐free island, the low calf survival is an important concern for population management. Potential management actions include increasing the supply of forage availability, either through habitat improvement or by providing supplementary forage. Milner et al. (2013) found that females that had access to supplementary forage in areas with low forage availability had lower winter mass loss than females without access to supplementary forage. The improved postwinter condition would subsequently have carryover affects to summer calf survival (Milner et al., 2013). However, managers would also need to consider other consequences of supplementary feeding, such as changing vegetation dynamics around feeding stations with cascading effects to other trophic levels (Milner, Van Beest, Schmidt, Brook, & Storaas, 2014). Although we found a habitat–performance relationship, further research is also needed to understand whether the advancement of spring and other climate factors are impacting the fitness of individuals and females’ ability to care for the calf, for example, during lactation.

## Conflict of Interest

None declared.
