# Supplementary figures and images for: Habitat–performance relationships of a large mammal on a predator‐free island dominated by humans
Source: Ecol Evol. 2016 Dec 20;7(1):305–19. doi: 10.1002/ece3.2594 (PMC5216668; doi:10.1002/ece3.2594)

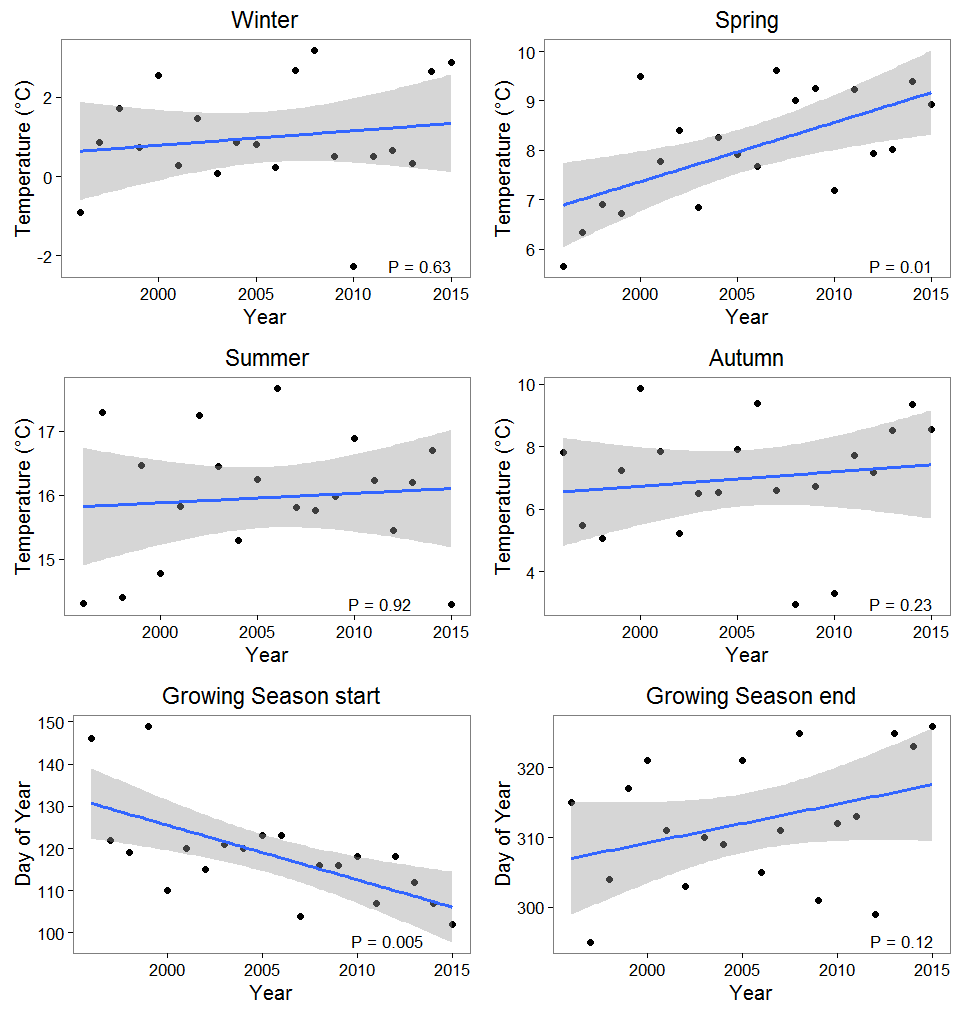

Supplement: Supplementary file 1 [file ECE3-7-305-s001.png]
