# Supplementary material for: Habitat–performance relationships of a large mammal on a predator‐free island dominated by humans
Source: Ecol Evol. 2016 Dec 20;7(1):305–19. doi: 10.1002/ece3.2594 (PMC5216668; doi:10.1002/ece3.2594)
Supplement: Supplementary file 2 [file ECE3-7-305-s002.docx]

**Appendix 2 –** The average proportion of habitats within the home ranges of moose in the north (n = 28) and the south (n = 17) of Öland for a) summer and b) winter. The habitat composition of the entire island is shown in c) for the north, south and the entire island (Total).

**
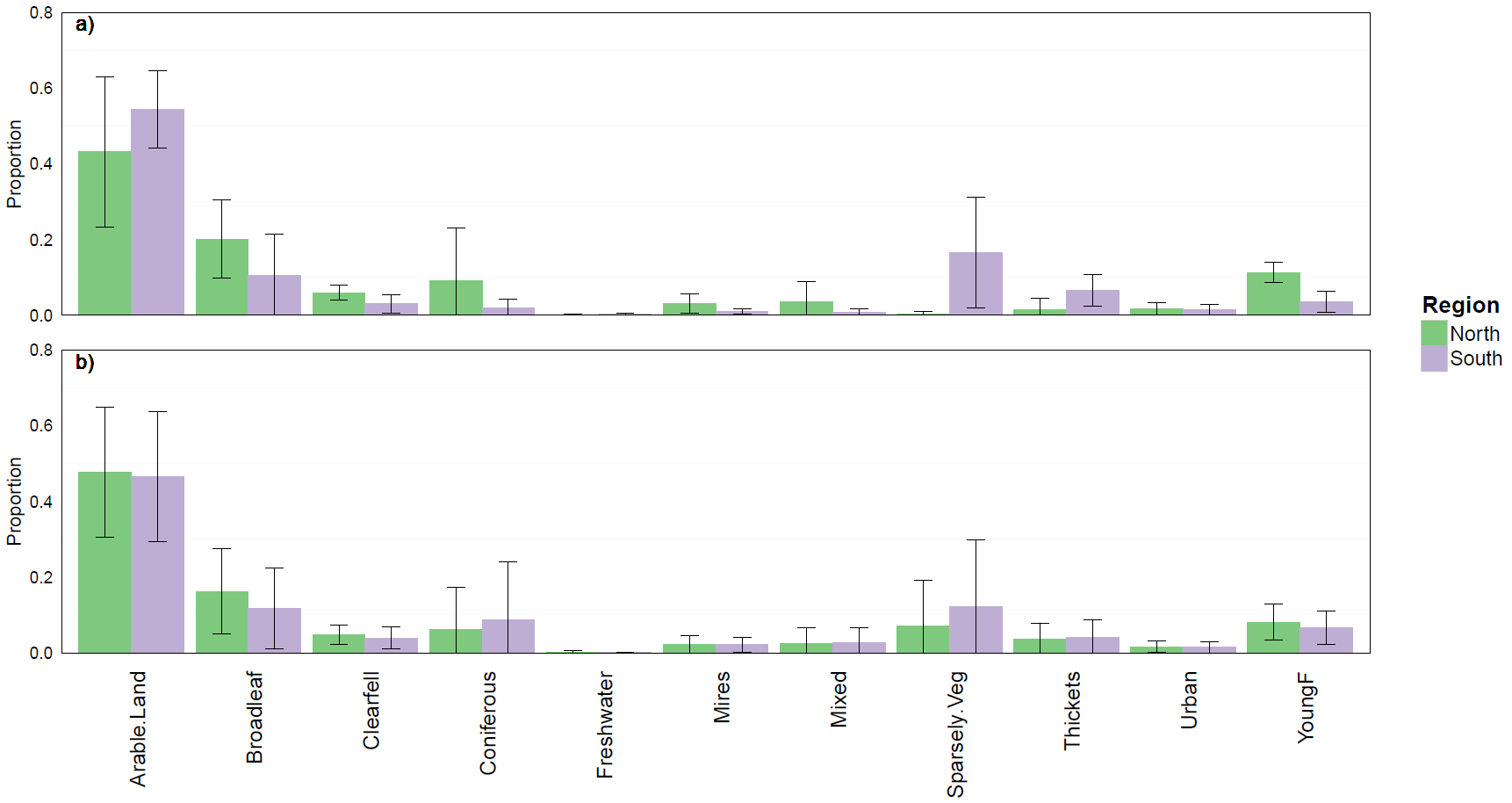

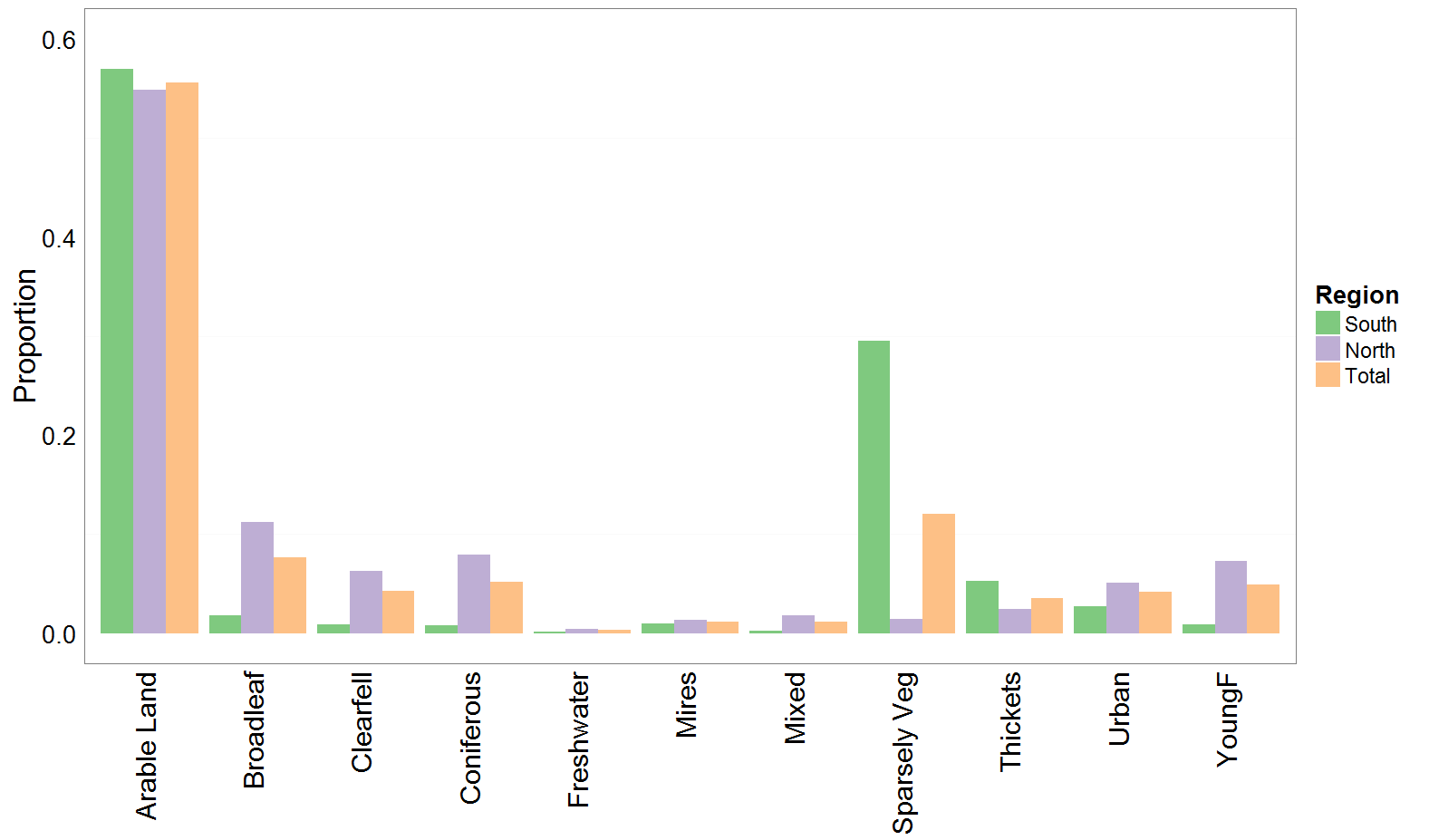
**

c)
