# Supplementary material for: Habitat–performance relationships of a large mammal on a predator‐free island dominated by humans
Source: Ecol Evol. 2016 Dec 20;7(1):305–19. doi: 10.1002/ece3.2594 (PMC5216668; doi:10.1002/ece3.2594)
Supplement: Supplementary file 3 [file ECE3-7-305-s003.docx]

**Appendix 3 – Supplementary results for the analysis of calving rates**

**
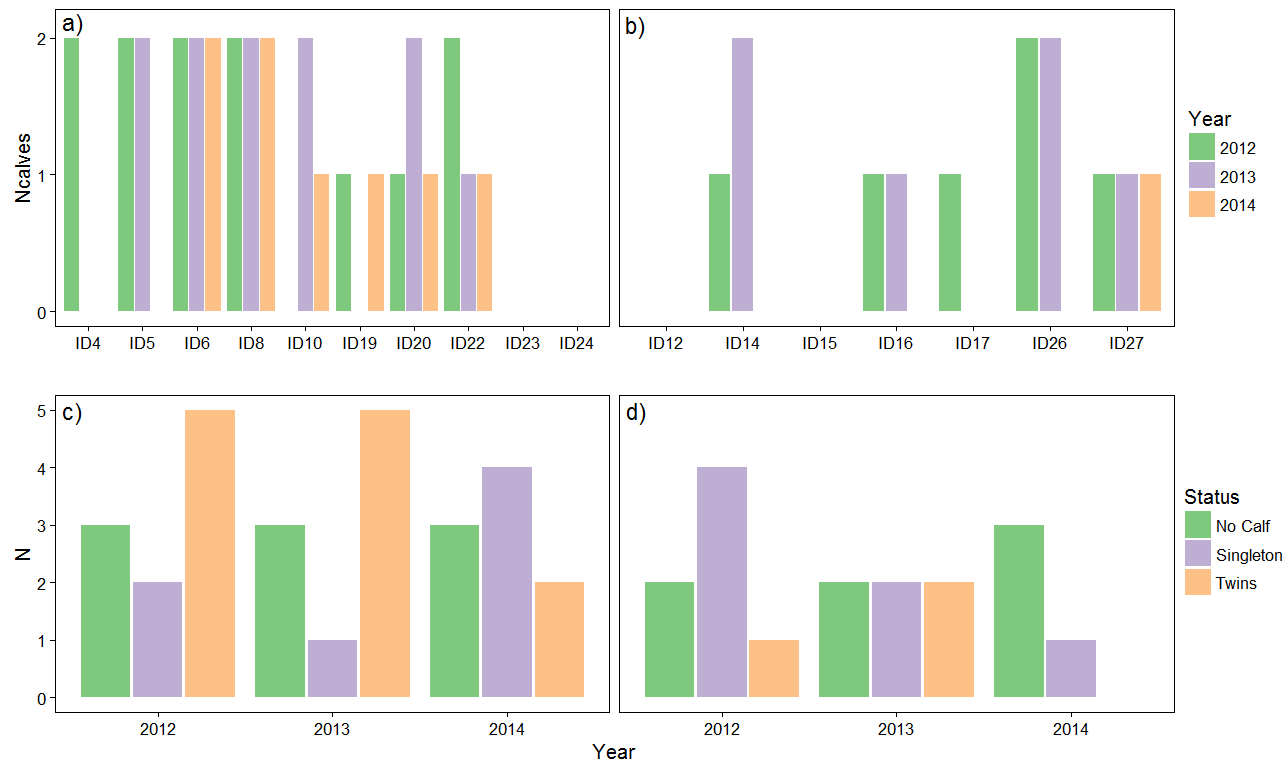
**

**Figure S1 –** Calving rates by individual (a,b) for the north (a) and south (b) of Öland. Calving rates are also shown by year for the north (c) and south (d).

**
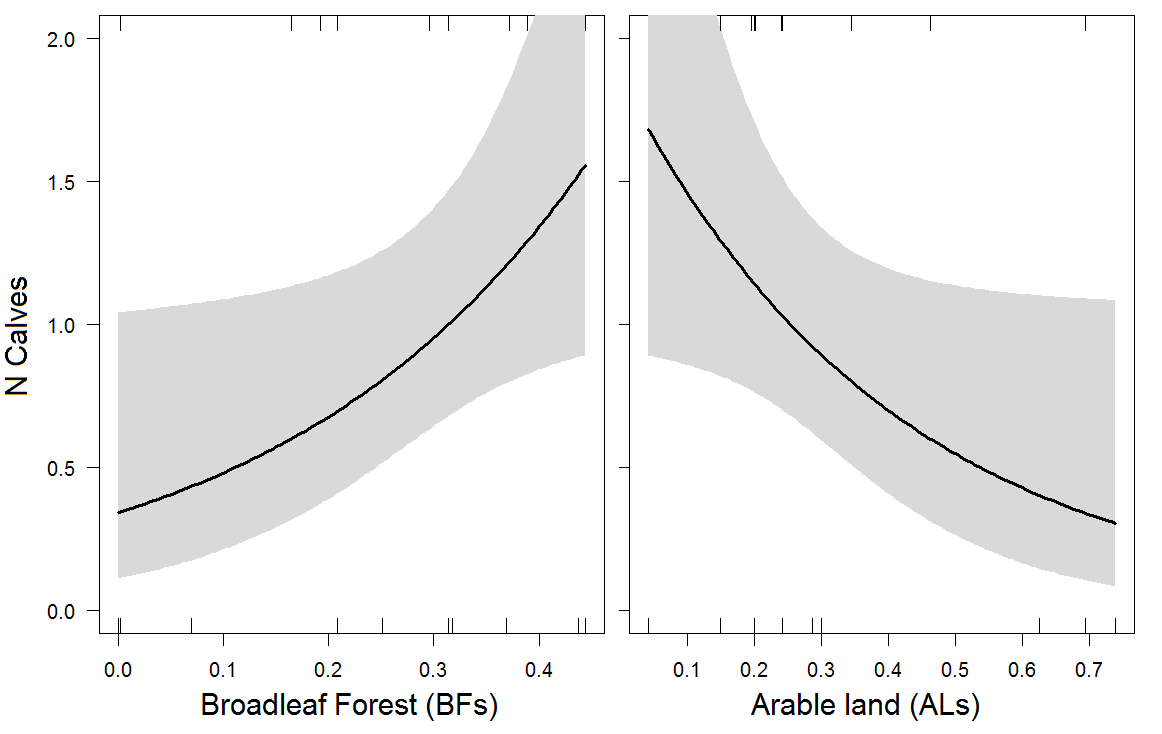
**

**Figure S2 –** Relationship for the top two models explaining variation in fecundity and use of broadleaf forest in summer (BFS, left) and Arable land in summer (ALs, right). The response is the number of calves (0, 1 or 2) and the explanatory variables are the percentage of time spent in each habitat modelled with a poisson distribution.

**Table S1 –** Model-averaged co-efficients for the variables included in all models with ΔAIC <2 (n = 5) that explain variation in fecundity. RVI is the relative variable importance, BF = broadleaf forest, AL = arable land, MX = mixed forest, CL = clearcut and s denotes the use during summer.

| Variable | Estimate | St.Error | Z value | P | RVI |
| --- | --- | --- | --- | --- | --- |
| BFs | 1.335 | 1.969 | 0.665 | 0.506 | 0.56 |
| ALs | -1.428 | 1.772 | 0.790 | 0.430 | 0.44 |
| MXs | -0.722 | 2.296 | 0.307 | 0.759 | 0.16 |
| CLs | 1.373 | 4.131 | 0.358 | 0.743 | 0.13 |
