# Supplementary material for: Habitat–performance relationships of a large mammal on a predator‐free island dominated by humans
Source: Ecol Evol. 2016 Dec 20;7(1):305–19. doi: 10.1002/ece3.2594 (PMC5216668; doi:10.1002/ece3.2594)
Supplement: Supplementary file 4 [file ECE3-7-305-s004.docx]

**Appendix 4** – Supplementary results for the logistical regression of factors affecting calf survival.

**Table S1** – Model-averaged coefficients of all variables included in Table 3. RVI is the relative variable importance and is estimated from all possible model combinations of the supplied variable, with the constraint of maximum 3 variables in a model. AL = arable land, CL = clear-cut, MI = Mires, MX = mixed forest, SV = sparsely vegetated area, TH = thicket, YF = young forest, s = summer and w = winter.

| Variable | Estimate | St.Error | Z-value | P | RVI |
| --- | --- | --- | --- | --- | --- |
| MIs | 58.382 | 38.416 | 1.453 | 0.146 | 0.55 |
| MXw | 70.923 | 53.05 | 1.291 | 0.197 | 0.49 |
| Twin | -2.947 | 1.815 | 1.553 | 0.12 | 0.49 |
| THw | -19.245 | 13.198 | 1.41 | 0.159 | 0.50 |
| CLs | -17.685 | 19.435 | 0.878 | 0.38 | 0.14 |
| ALw | -3.403 | 4.505 | 0.734 | 0.463 | 0.14 |
| MXs | -19.869 | 40.285 | 0.482 | 0.63 | 0.13 |
| SVw | 9.321 | 12.674 | 0.709 | 0.479 | 0.13 |
| YFw | 7.829 | 11.379 | 0.667 | 0.505 | 0.12 |
|  |  |  |  |  |  |
|  |  |  |  |  |  |
|  |  |  |  |  |  |
|  |  |  |  |  |  |
|  |  |  |  |  |  |
|  |  |  |  |  |  |
